# Supplementary material for: Regime shifts occur disproportionately faster in larger ecosystems
Source: Nat Commun. 2020 Mar 10;11:1175. doi: 10.1038/s41467-020-15029-x (PMC7064493; doi:10.1038/s41467-020-15029-x)
Supplement: Supplementary file 1 — Supplementary Information [file 41467_2020_15029_MOESM1_ESM.pdf]

# Supplementary Information

# Regime shifts occur disproportionately faster in larger ecosystems

Cooper et al.

## Contents

Supplementary Note 1. Linear model fit of the empirical data ........ 1

Supplementary Note 2. Omitted observations from modelling experiments ........ 1

Supplementary Note 3. Amendments to the Game of Life agent-based model code ........ 2

Supplementary Note 4. Amendments to the Language Change agent-based model code. ........ 3

Supplementary Note 5. Model code to run the SH model. ........ 4

Supplementary Tables ........ 5

Supplementary Figures ........ 20

Supplementary References ........ 33

## Supplementary Note 1. Linear model fit of the empirical data

Whilst the main manuscript finds that a significant positive and sublinear relationship exists between system size and regime shift duration, it is reasonable to ask the extent to which a linear fit would better capture the variance in the underlying dataset. Therefore, this section provides a comparative analysis of the empirical sub-linear trend by calculating a linear regression model from the unlogged (i.e. raw values) data, as well as its associated uncertainty and implications for the duration of real-life regime shifts.

We calculate a simple linear regression model between the 42 system areas and regime shifts (Supplementary Table 1) in the statistical software R[1]. As per the sub-linear trend, an overarching positive relationship exists between system area and regime shift duration (Supplementary Fig. 1), so that larger systems tend to take longer to shift between two stable regimes. The fit of the linear regression model is superior ($R^2 = 0.947$, $p < 0.001$, df = 40) to that of the sub-linear relationship ($R^2 = 0.491$, $p < 0.001$, df = 40) when all 42 data points are included. Using the linear model, we are able to derive a second set of projections for the durations of regime shifts across the Amazon rainforest and Caribbean coral reefs. For the Amazon, the linear model (Supplementary Fig. 1) produces a mean duration of 233 years (CI: 210-255 years), which sits in the upper end of our sub-linear uncertainty bounds. Likewise, for the Caribbean coral reefs, the linear model generates a mean duration of 10 years (CI: 5-14 years), which is five years quicker than the sub-linear projection.

However, it is important to note that unlike the sub-linear relationship ($R^2 = 0.423$, $p < 0.001$, df = 39), the linear relationship is not robust. The sub-linear relationship is present in all 42 alternative models (see Supplementary Table 2), whilst the linear relationship is only observed in 41 of the alternative models. Essentially, the linear relationship becomes insignificant as soon as the record for the Sahara is removed ($R^2 = 0.050$, $p > 0.1$, df = 39), potentially highlighting that the high $R^2$ value was due to overfitting to this datapoint. In contrast, the sub-linear relationship remains robust to the removal of individual data points (Supplementary Table 2) and random errors between 50-150% of the original duration values (Supplementary Figures 5 and 6). Thus, it is the robust, sub-linear models that we include in the main text.

## Supplementary Note 2. Omitted observations from modelling experiments

Supplementary table 9 records the number of model iterations that were omitted from the regression models displayed in Fig. 3. As detailed in the Methods, omitted runs did not reach a stable alternative state over the duration of their simulation. All model runs that demonstrated a regime shift (i.e. reached a stable alternative state) were included in our analyses.

# Supplementary Note 3. Amendments to the Game of Life agent-based model code

See Supplementary Table 5 for variable descriptions.

The altered code was as follows:

```
to go
    ask patches
        [if range = 4 [ set live-neighbors count neighbors4 with [living?]]] ;; if range is 4
        then each patch looks at 4 neighbor patches
     ask patches
         [if range = 8 [ set live-neighbors count neighbors with [living?]]] ;; if range is 8
        then each patch looks at 8 neighbor patches
     ;; Starting a new "ask patches" here ensures that all the patches
    ;; finish executing the first ask before any of them start executing
    ;; the second ask.  This keeps all the patches in synch with each other,
    ;; so the births and deaths at each generation all happen in lockstep.
    ask patches
        [ ifelse live-neighbors = (range / 2) ;; whatever the range, this is done for the same
        proportion of neighbor patches
            [ cell-birth ]
            [ if live-neighbors != (range / 4) ;; whatever the range, this is done for the
            same proportion of neighbor patches
            [ cell-death ] ] ]
    stop-function
    ifelse stability-score = 100
    [stop] ;; stop the model when the stability score is 100 (i.e. number of alive patches has
    not changed for 100 time-steps
    [tick]
end

to stop-function
    let alive-now (count patches with [pcolor = fgcolor]) ;; how many patches are alive?
      ifelse (alive-now = total-live) ;; if the number of alive patches is the same as the last time-
    step, then add one to the stability score
        [set stability-score (stability-score + 1)]
        [set stability-score 0]
    set total-live (alive-now)
end
```

## Supplementary Note 4. Amendments to the Language Change agent-based model code.

See Supplementary Table 6 for variable descriptions.

The altered code was as follows:

```
to create-network
    ;; make the initial network of two nodes and an edge
    let partner nobody
    let first-node one-of nodes
    let second-node one-of nodes with [self != first-node]
    ;; make the first edge
    ask first-node [ create-link-with second-node [ set color white ] ]
    ;; randomly select unattached node to add to network
    let new-node one-of nodes with [not any? link-neighbors]
    ;; and connect it to a partner already in the network
    while [new-node != nobody] [
        set partner find-partner
        ask new-node [ create-link-with partner [ set color white ] ]
        layout
        set new-node one-of nodes with [not any? link-neighbors]
    ]
    ;; The above code is present in the standard NetLogo LC model. It ensures all nodes are in
    the network. The below additions determine how many extra links (determined by num-
    connections) are added on top of this.
    if num-connections < (num-nodes - 1) [set num-connections (num-nodes - 1)]
    ;; stops the number of connections being too small
    while [count links < num-connections] [
        let first-nodeB one-of nodes
        let second-nodeB one-of nodes with [self != first-nodeB]
        ask first-nodeB [create-link-with second-nodeB [ set color white ] ]
        ;; find a node, find a different node and link them if you need more links
        layout
    ]
    ;; while more connections are required, they are continually added
    if count (links) > num-connections [create-network]
    ;; if there are have too many connections the process is restarted
end
```

## Supplementary Note 5. Model code to run the SH model.

See Supplementary Table 7 for variable descriptions.

```
to go
	set Zcons ((g * Z * A1) / (A1 + h))
	set A1_increase ((r * A1 * (1 - (A1 / K))) - Zcons + (( d / f)*(A2 - A1)))
	set A2_increase ((r * A2 *(1 - (A2 / K))) - ((d /(1 - f))*(A1 - A2)))
	set Z_increase ((Zcons * ez) - (m * Z))
	ifelse A1 > 0
		[set A1 (A1 + A1_increase)]
		[set A1 0]
	ifelse A2 > 0
		[set A2 (A2 + A2_increase)]
		[set A2 0]
	ifelse Z > 0
		[set Z (Z + Z_increase)]
		[set Z 0]
	tick
end
```

## Supplementary Tables

**Supplementary Table 1|** Details of the empirical regime shifts depicted in Fig. 2 (record numbers 1-42) and Supplementary Fig. 2 (record numbers 1-50). Unless estimated or originally reported as an integer, system surface areas, depths and shift durations are rounded to a maximum of 3 decimal places. The system volumes were calculated by multiplying the surface areas by the mean depths. System depths and volumes shown as a dash (-) could not be reliably estimated.

| System | Record number | Case study | System surface area ($km^2$) | System mean depth (m) | System volume (million $m^3$) | Regime shift duration (years) | Initial regime | Alternate regime | Reference |
|---|---|---|---|---|---|---|---|---|---|
| Freshwater | 1 | Lake Erhai, China | 250 | 11 | 2750 | 2 | Oligotrophic | Eutrophic | [2,3] |
| | 2 | Paul and Peter Lakes, USA | 0.020 | 8.30 | 0.199 | 0.077 | Undisturbed food web state | Predated food web state | [4,5] |
| | 3 | Lake Veluwe, Netherlands | 30 | 1.30 | 39 | 2 | Oligotrophic | Eutrophic | [6,7] |
| | 4 | Mwanza gulf, Lake Victoria, Tanzania | 500 | 40 | 20,000 | 8 | Herbivore dominated | Small predators dominated | [6,7] |
| | 5 | Lake Krankesjön, Sweden | 2.90 | 1.50 | 6.30 | 3 | Turbid | Oligotrophic | [8] |

| | | | | | | | | | |
|---|---|---|---|---|---|---|---|---|---|
| | 6 | Lake Stechlin, Germany | 4.23 | 20 | 84.6 | 0.030 | Oligotrophic | Eutrophic | [9] |
| | 7 | Old Danube Lake, Austria | 1.60 | 2.30 | 3.68 | 5 | Oligotrophic | Eutrophic | [10] |
| | 8 | Lake Kariba, Zimbabwe/ Zambia | 5400 | 29 | 156,600 | 29 | Productive fishery | Collapsed fishery | [11] |
| | 9 | Lake of the woods, Canada/USA | 4350 | 3.40 | 14,790 | 35 | *Aulacoseira subarctica* diatom dominance | *Cyclotella* spp. diatom dominance | [12] |
| | 10 | Foy Lake, USA | 1.95 | - | - | 5 | Low benthic : planktonic diatom ratio | High benthic : planktonic diatom ratio | [13] |
| | 11 | Lake Chilika, India | 1000 | 1.50 | 1500 | 8 | Productive fishery | Collapsed fishery | [14] |
| | 12 | Lac de Tunis, Tunisia | 48.6 | 1.50 | 72.9 | 1 | Productive fishery | Collapsed fishery | [15] |
| | 13 | Victoria Park lake, Australia | 0.15 | 1.10 | 0.165 | 0.29 | Oligotrophic | Eutrophic | [16] |
| Marine | 14 | Jamaican coral reef | 1000 | - | | 15 | Coral reef dominance | Macroalga dominance | [17,18] |

| | | | | | | | | |
|---|---|---|---|---|---|---|---|---|
| 15 | Black sea | 436,402 | 1200 | $5.24 \times 10^8$ | 40 | Large predator dominated | Depleted large predators | [19] |
| 16 | Northern Gulf of Alaska | 1250 | - | - | 17 | Shrimp dominated | Depleted shrimp populations | [20,21] |
| 17 | Northern Gulf of Mexico | 18,000 | 1600 | $2.88 \times 10^7$ | 15 | Pre-hypoxic | Hypoxic | [22,23] |
| 18 | Florida Bay, USA | 40 | 1.50 | 60 | 5 | Sea-grass dominated | Algal eutrophic conditions | [24,25] |
| 19 | Ringkøbing Fjord, Denmark | 300 | 3 | 900 | 3 | Turbid state | Clear water state | [26] |
| 20 | Frisian Front, Germany | 2880 | 50 | 144,000 | 5 | Brittle star dominated | Mud shrimp dominated | [27] |
| 21 | Eastern Scotian Shelf | 108,000 | 90 | $9.72 \times 10^6$ | 5 | Productive fishery | Collapsed fishery | [28] |
| 22 | Gulf of Trieste | 600 | 19 | 11,400 | 3 | Annual red tide events | Repeated mucilage events | [29,30] |
| 23 | Central Baltic sea | 100,000 | 70 | $2.64 \times 10^7$ | 5 | High ecosystem state index | Collapsed ecosystem state index | [31,32] |

| | | | | | | | | |
|---|---|---|---|---|---|---|---|---|
| 24 | East Florida Mangroves, USA | 20 | 10 | 170 | 4 | Salt marshes | Mangrove stands | [33] |
| 25 | Newfoundland, Canada | 400,000 | 50 | $2.00 \times 10^7$ | 28 | Productive fishery | Collapsed fishery | [34] |
| 26 | Northern Benguela marine system | 24,000 | - | - | 15 | Productive fishery | Collapsed fishery | [35] |
| 27 | Chesapeake Bay, USA | 11,600 | 7 | 81,200 | 23 | Productive fishery | Collapsed fishery | [36,37] |
| 28 | Aldabra atoll | 115 | 5 | 575 | 1 | Scleractinian coral dominance | Soft coral dominance | [38,39] |
| 29 | Alamitos Bay, USA | 820 | - | - | 0.96 | Stable fishery biomass | Collapsed fishery biomass | [40] |
| 30 | Kongsfjorden, Svalbard | 230 | - | - | 2 | Low species diversity index | High species diversity index | [41,42] |
| 31 | Izmit Bay, Turkey | 310 | 1.80 | 540 | 1 | Stable meiofauna community | Collapsed meiofauna community | [43] |

| | | | | | | | | | |
|---|---|---|---|---|---|---|---|---|---|
| | 32 | Mariager fjord, Denmark | 20 | 30 | 600 | 0.04 | Stable macrofauna community | Collapsed macrofauna community | [44] |
| | 33 | Gulf of Finland | 5700 | 38 | 217,000 | 2 | Stable faunal community | Collapsed faunal community | [45] |
| | 34 | Georges Bank, USA/Canada | 43,000 | 65 | $2.80 \times 10^6$ | 19 | Stable haddock fishery | Collapsed haddock fishery | [46] |
| | 35 | Nova Scotia kelp beds, Canada | 140 | - | - | 6.00 | Kelp dominated ecosystem | Urchin dominated ecosystem | [47] |
| | 36 | Peruvian anchovy fishery | 14,000 | - | - | 14.00 | Growing anchovy fishery | Collapsed anchovy fishery | [48] |
| | 37 | Limfjorden, Denmark | 1500 | 6.5 | 9750 | 13.00 | Growing oyster fishery | Collapsed oyster fishery | [49] |
| | 38 | Osaka Bay ostracods, Japan | 1400 | 28 | 39,200 | 50.00 | Stable Ostracod population | Collapsed Ostracod population | [50] |
| Terrestrial | 39 | The Sahel | 9,400,000 | 1 | $9.40 \times 10^6$ | 400 | Vegetated Sahel | Desertified Sahel | [51] |
| | 40 | Maradi agri-system, Niger | 35,100 | 10 | 351,000 | 20 | Agriculture | Desert | [52] |

| | | | | | | | | | |
|---|---|---|---|---|---|---|---|---|---|
| | 41 | Zion National Park, Utah, USA | 4500 | 10 | 45,000 | 8 | Woody vegetation dominated | Native species collapse | [53] |
| | 42 | Chobe National Park, Botswana | 11,700 | 10 | 117,000 | 15 | Savanna grassland | Woodland | [54] |
| Social | 43 | Babylon | 500,000 | - | - | 23 | Functioning civilisation | Collapsed civilisation | [55] |
| | 44 | Hittite Empire | 450,000 | - | - | 500 | Functioning civilisation | Collapsed civilisation | [55] |
| | 45 | Western Chous dynasty | 500,000 | - | - | 163 | Functioning civilisation | Collapsed civilisation | [57,58] |
| | 46 | Roman | 4,140,000 | - | - | 432 | Functioning civilisation | Collapsed civilisation | [56] |
| | 47 | Assyria | 650,000 | - | - | 217 | Functioning civilisation | Collapsed civilisation | [55] |
| | 48 | Harappan civilization | 250,000 | - | - | 300 | Functioning civilisation | Collapsed civilisation | [55] |
| | 49 | Mayan | 324,000 | - | - | 100 | Functioning civilisation | Collapsed civilisation | [57] |
| | 50 | Almoravid-Almohad | 2,300,000 | - | - | 122 | Functioning civilisation | Collapsed civilisation | [58] |

**Supplementary Table 2|** Details of the first sensitivity analysis experiment described in Supplementary Note 2. Here, 42 alternative regression models were created by removing one of the empirical records (df = 39 for each alternative model). The 'original model' refers to the model detailed in Fig. 2.

| Alt. model number | Case study removed from alternative model | Model slope (b-term) | Significance (p-value) |
|---|---|---|---|
| N/A | None – this is the full model displayed in Fig. 2 | 0.221 | $1.31 \times 10^{-7}$ |
| 1 | The Sahel | 0.189 | $2.62 \times 10^{-6}$ |
| 2 | Central Baltic sea | 0.235 | $3.68 \times 10^{-8}$ |
| 3 | East Floridia Mangroves, USA | 0.234 | $1.85 \times 10^{-8}$ |
| 4 | Foy Lake, USA | 0.233 | $7.40 \times 10^{-8}$ |
| 5 | Old Danube Lake, Austria | 0.232 | $8.26 \times 10^{-8}$ |
| 6 | Eastern Scotian Shelf | 0.229 | $6.59 \times 10^{-8}$ |
| 7 | Northern Gulf of Alaska | 0.229 | $1.60 \times 10^{-7}$ |
| 8 | Lake Stechlin, Germany | 0.213 | $4.68 \times 10^{-7}$ |
| 9 | Mariager fjord, Denmark | 0.213 | $3.14 \times 10^{-7}$ |
| 10 | Paul and Peter Lakes, USA | 0.216 | $6.19 \times 10^{-7}$ |
| 11 | Lake Krankesjön, Sweden | 0.225 | $1.79 \times 10^{-7}$ |
| 12 | Black sea | 0.217 | $4.23 \times 10^{-7}$ |
| 13 | Lac de Tunis, Tunisia | 0.217 | $2.62 \times 10^{-7}$ |
| 14 | Aldabra atoll | 0.217 | $2.17 \times 10^{-7}$ |
| 15 | Victoria Park lake, Australia | 0.217 | $5.29 \times 10^{-7}$ |
| 16 | Gulf of Finland | 0.224 | $8.28 \times 10^{-8}$ |
| 17 | Florida Bay, USA | 0.223 | $1.65 \times 10^{-7}$ |
| 18 | Lake of the woods, Canada/USA | 0.218 | $1.41 \times 10^{-7}$ |
| 19 | Izmit bay, Turkey | 0.218 | $1.65 \times 10^{-7}$ |
| 20 | Lake Kariba, Zimbabwe/Zambia | 0.218 | $1.70 \times 10^{-7}$ |
| 21 | Chesapeake Bay, USA | 0.218 | $2.18 \times 10^{-7}$ |
| 22 | Kongsfjorden, Svalbard | 0.219 | $2.03 \times 10^{-7}$ |
| 23 | Lake Erhai, China | 0.219 | $2.00 \times 10^{-7}$ |
| 24 | Maradi agri-system, Niger | 0.219 | $2.48 \times 10^{-7}$ |
| 25 | Nova Scotia kelp beds, Canada | 0.222 | $1.73 \times 10^{-7}$ |
| 26 | Alamitos Bay, USA | 0.220 | $1.16 \times 10^{-7}$ |
| 27 | Lake Veluwe, Netherlands | 0.220 | $2.51 \times 10^{-7}$ |
| 28 | Ringkøbing Fjord, Denmark | 0.220 | $2.01 \times 10^{-7}$ |
| 29 | Chobe National Park, Botswana | 0.220 | $2.15 \times 10^{-7}$ |
| 30 | Jamaican coral reef | 0.221 | $2.21 \times 10^{-7}$ |
| 31 | Georges Bank, USA/Canada | 0.220 | $2.41 \times 10^{-7}$ |
| 32 | Gulf of Trieste | 0.220 | $1.84 \times 10^{-7}$ |
| 33 | Frisian Front, Germany | 0.221 | $1.69 \times 10^{-7}$ |
| 34 | Northern Gulf of Mexico | 0.220 | $2.20 \times 10^{-7}$ |
| 35 | Peruvian anchovy fishery | 0.220 | $2.14 \times 10^{-7}$ |
| 36 | Newfoundland, Canada | 0.220 | $3.29 \times 10^{-7}$ |
| 37 | Mwanza gulf, Lake Victoria, Tanzania | 0.221 | $1.86 \times 10^{-7}$ |
| 38 | Northern Benguela marine system | 0.220 | $2.22 \times 10^{-7}$ |
| 39 | Osaka Bay ostracods, Japan | 0.220 | $5.68 \times 10^{-8}$ |
| 40 | Zion National Park, Utah, USA | 0.221 | $1.87 \times 10^{-7}$ |
| 41 | Limfjorden, Denmark | 0.220 | $1.73 \times 10^{-7}$ |
| 42 | Lake Chilika, India | 0.221 | $1.85 \times 10^{-7}$ |

**Supplementary Table 3|** The parameters of the Wolf-Sheep Predation agent-based model for the default set-up in NetLogo and the world size experiment.

| Experiment | Variable | Description | Default value |
|---|---|---|---|
| Default (simulation outputs not presented here) | World Size | The number of pixels that make up the length and width of the landscape | 50 x 50 pixels |
| | Grass Setting | The setting to select the variant of the WSP model which includes grass | On |
| | Grass regrowth time | The number of model steps required for grass to regrow after being consumed | 30 steps |
| | Initial number wolves | The initial number of wolves | 50 |
| | Wolf gain from food | The energy gained by wolves after consuming a sheep | 20 |
| | Sheep gain from food | The energy gained by sheep after consuming grass | 4 |
| | Wolf reproduce | The probability each wolf has of reproducing every time-step | 5 % |
| | Sheep reproduce | The probability each sheep has of reproducing every time-step | 4 % |
| | Move | The number of pixels animals can move each time step in a random direction | 1 |
| 1.1. Effect of system size on shift duration | World Size | The number of pixels that make up the length and width of the landscape | 1-100 x 1-100 pixels |
| | Grass Setting | The setting to select the variant of the WSP model which includes grass | On |
| | Grass regrowth time | The number of model steps required for grass to regrow after being consumed | 1-100 |
| | Initial number wolves | The initial number of wolves | World size / 50 |
| | Initial number sheep | The initial number of sheep | World size / 25 |
| | Wolf gain from food | The energy gained by wolves after consuming a sheep | 20 |
| | Sheep gain from food | The energy gained by sheep after consuming grass | 4 |
| | Wolf reproduce | The probability each wolf has of reproducing every time-step | 5 % |
| | Sheep reproduce | The probability each sheep has of reproducing every time-step | 7 % |
| | Move | The number of pixels animals are able to move each time step in a random direction | 1 |

**Supplementary Table 4|** The parameters of the Wolf-Sheep Predation agent-based model for the modularity and fluidity experiments.

| **Experiment** | **Variable** | **Description** | **Default value** |
|---|---|---|---|
| 1.2 Effect of system modularity on shift duration | World Size | The number of pixels that make up the length and width of the landscape | (2, 5 10, 20, 50, or 100) x 100 pixels |
| | Grass Setting | The setting to select the variant of the WSP model which includes grass | On |
| | Grass regrowth time | The number of model steps required for grass to regrow after being consumed | 1-100 |
| | Initial number wolves | The initial number of wolves | World size / 50 |
| | Initial number sheep | The initial number of sheep | World size / 25 |
| | Wolf gain from food | The energy gained by wolves after consuming a sheep | 20 |
| | Sheep gain from food | The energy gained by sheep after consuming grass | 4 |
| | Wolf reproduce | The probability each wolf has of reproducing every time-step | 5 % |
| | Sheep reproduce | The probability each sheep has of reproducing every time-step | 7 % |
| | Move | The number of pixels animals are able to move each time step in a random direction | 1 |
| 1.3 Effect of system fluidity on shift duration | World Size | The number of pixels that make up the length and width of the landscape | 100 x 100 pixels |
| | Grass Setting | The setting to select the variant of the WSP model which includes grass | On |
| | Grass regrowth time | The number of model steps required for grass to regrow after being consumed | 1-100 |
| | Initial number wolves | The initial number of wolves | 204 |
| | Initial number sheep | The initial number of sheep | 408 |
| | Wolf gain from food | The energy gained by wolves after consuming a sheep | 20 |
| | Sheep gain from food | The energy gained by sheep after consuming grass | 4 |
| | Wolf reproduce | The probability each wolf has of reproducing every time-step | 5 % |
| | Sheep reproduce | The probability each sheep has of reproducing every time-step | 7 % |
| | Move | The number of pixels animals are able to move each time step in a random direction | 1-100 |

**Supplementary Table 5|** The parameters of the Game of Life model for the default set-up and world size, system modularity and fluidity experiments.

| Experiment | Variable | Description | Default Value |
|---|---|---|---|
| Default | World size | The number of pixels that make up the length and width of the landscape | 100 x 100 pixels |
| | Initial density | Determines the initial density of cells that are alive. These are randomly placed during setup. | 35% |
| 2.1 Effect of system size on regime shift duration | World size | The number of pixels that make up the length and width of the landscape | 1-100 x 1-100 pixels |
| | Initial density | Determines the initial density of cells that are alive. These are randomly placed during setup. | 35% |
| 2.2 Effect of system modularity on regime shift duration | World size | The number of pixels that make up the length and width of the landscape | (2, 5, 10, 20, 50 or 100) x 1-100 pixels |
| | Initial density | Determines the initial density of cells that are alive. These are randomly placed during setup. | 35% |
| 2.3 Effect of fluidity on regime shift duration | World size | The number of pixels that make up the length and width of the landscape | 1-100 x 1-100 pixels |
| | Initial density | Determines the initial density of cells that are alive. These are randomly placed during setup. | 35% |
| | Range | This is a new variable to determine how many neighbouring cells are considered by each cell during the decision to live or die. | 4 or 8<br>The latter is equivalent to the default model. |

**Supplementary Table 6|** The parameters of the Language Change model for the default set-up, and the number of nodes and number of connections experiments. The parameter values for experiment 3.3 (heterogeneity of connections) are also valid for experiment 3.2 (number of connections).

| Experiment | Variable | Description | Default value |
|---|---|---|---|
| Default | Number of nodes | The number nodes included in the model | 100 |
| | Percent grammar 1 | The percentage of nodes with language 1 | 60 % |
| | Update algorithm | Select which variant of the model you choose to run | Individual |
| | Sink state 1 | Once an individual adopts language 1, it cannot go back | On |
| 3.1. Effect of the number of nodes on shift duration | Number of nodes | The number nodes included in the model | 3-1,000 |
| | Number of connections | The number of connections within the system | (Number of nodes – 1) |
| | Percent grammar 1 | The percentage of nodes with language 1 | 60 % |
| | Update algorithm | Select which variant of the model you choose to run | Individual |
| | Sink state 1 | Once an individual adopts language 1, it cannot go back | On |
| 3.3. Effect of connection heterogeneity on shift duration | Number of nodes | The number nodes included in the model | 100 |
| | Number of connections | The number of connections within the system | 99-4,500 |
| | Percent grammar 1 | The percentage of nodes with language 1 | 60 % |
| | Update algorithm | Select which variant of the model you choose to run | Individual |
| | Sink state 1 | Once an individual adopts language 1, it cannot go back | On |

**Supplementary Table 7|** The parameters of the Spatial Heterogeneity predator-prey model for the default set-and the two spatial scale experiments.

| Experiment | Variable | Description | Default value |
|---|---|---|---|
| Default | A | Concentration of algae | 8 |
| | A1 | Proportion of algae inside | 0.8 |
| | A2 | Proportion of algae outside | 1 – A1 |
| | D | Fraction of volume exchanged between parts inside and outside | 0.01 |
| | Ez | Efficiency of conversion of food into growth of zooplankton | 0.6 |
| | F | | 0.5 |
| | G | Maximum grazing rate of zooplankton | 0.4 |
| | H | Half saturation concentration of algae for Z functional response | 0.6 |
| | K | Carrying capacity of algae | 10 |
| | Q | Fraction of total lake volume occupied by zooplankton (i.e. inside) | A1 |
| | M | Mortality rate of zooplankton | 0.15 |
| | R | Maximum growth rate of algae | 0.5 |
| | Z | Concentration of large herbivorous zooplankton | A / 4 |
| | Zcons | | 0 |
| 5.1 Effect of system size (K) on regime shift duration | A | Concentration of algae | 8 |
| | A1 | Proportion of algae inside | 0.8 |
| | A2 | Proportion of algae outside | 1 – A1 |
| | D | Fraction of volume exchanged between parts inside and outside | 0.01 |
| | Ez | Efficiency of conversion of food into growth of zooplankton | 0.6 |
| | F | | 0.5 |
| | G | Maximum grazing rate of zooplankton | 0.4 |
| | H | Half saturation concentration of algae for Z functional response | 0.6 |
| | K | Carrying capacity of algae | 1-100 |
| | Q | Fraction of total lake volume occupied by zooplankton (i.e. inside) | A1 |
| | M | Mortality rate of zooplankton | 0.15 |
| | R | Maximum growth rate of algae | 0.5 |
| | Z | Concentration of large herbivorous zooplankton | A / 4 |
| | Zcons | | 0 |
| 5.2 Effect of fluidity (d) on regime shift duration | A | Concentration of algae | 8 |
| | A1 | Proportion of algae inside | 0.8 |
| | A2 | Proportion of algae outside | 1 – A1 |
| | D | Fraction of volume exchanged between parts inside and outside | 0-1 |
| | Ez | Efficiency of conversion of food into growth of zooplankton | 0.6 |

| | | |
|---|---|---|
| F | | 0.5 |
| G | Maximum grazing rate of zooplankton | 0.4 |
| H | Half saturation concentration of algae for Z functional response | 0.6 |
| K | Carrying capacity of algae | 10 |
| Q | Fraction of total lake volume occupied by zooplankton (i.e. inside) | A1 |
| M | Mortality rate of zooplankton | 0.15 |
| R | Maximum growth rate of algae | 0.5 |
| Z | Concentration of large herbivorous zooplankton | A / 4 |
| Zcons | | 0 |

**Supplementary Table 8|** The regression coefficients of the linear models describing the association between the different spatial properties and the duration of system shift. These models are created from the raw (unlogged) data, with the final column of the table comparing the strength of these linear models to the strength of the nonlinear models presented in the main manuscript (see Fig. 2). Significance levels: '-' p > 0.05; 0.05 > '*' ≥ 0.01; 0.01 > '**' ≥ 0.001; 0.001 > p '***'.

| Model name | Model type | Parameter varied | Model slope (b-term) | Coefficient of determination ($R^2$) | Significance (p-value) | Degrees of freedom (df) |
|---|---|---|---|---|---|---|
| Wolf- Sheep -Predation (WSP) | Agent-based | (1.1) Model total area | 0.53 | 0.04 | *** | 179,096 |
| | | (1.2) Module size (divide constant 100 x 100 area into sub-worlds) | 49.0 | 0.23 | *** | 19,121 |
| | | (1.3) Maximum distance wolves and sheep can move per timestep | 0.43 | 0.00 | - | 7177 |
| Game of Life (GoL) | Cellular automata | (2.1) Model total area | 0.25 | 0.21 | *** | 246,235 |
| | | (2.2) Module size (divide constant 100 x 100 area into discrete sub-worlds) | 127 | 0.51 | *** | 598 |
| | | (2.3) Number of neighbouring cells any one cell can interact with | -5635 | 0.63 | *** | 102 |
| Language Change (LC) | Network-structured | (3.1) Number of network nodes | 0.00 | 0.00 | - | 99,838 |
| | | (3.2) Number of inter-nodal connections | 0.00 | 0.00 | - | 440,198 |
| | | (3.3) Standard deviation of connections measured from experiment 3.2. | 0.97 | 0.27 | *** | 440,198 |
| Lake Chilika fishery (LCH) | System dynamics model | (4) Model total area | 0.01 | 0.89 | *** | 2751 |
| Spatial Heterogeneity (SH) | Ordinary differential equation | (5.1) Carrying capacity for algae (i.e. model size) | 0.18 | 0.97 | *** | 92 |
| | | (5.2) Fraction of volume exchanged between model parts (i.e. diffusion of stress) | -14.9 | 0.22 | *** | 84 |

**Supplementary Table 9|** The omitted model runs expressed as an absolute number and as the percentage of total model runs per experiment. †In GoL-2.2, the two simulations with the highest degrees of modularity (e.g. module sizes '2' and '5') did not record any shifts. Therefore, any model runs in GoL-2.2 that did not record regime shifts over the 5000 timesteps were given a regime shift duration of 5001 time-steps. This conservative estimate ensured that results from all six module sizes could be included in the analysis.

| **Experiment number** | **Parameter varied** | **Number of omitted model runs** | **Proportion of total model runs omitted (%)** |
|---|---|---|---|
| WSP-1.1 | Model total area | 80,912 | 31.1 |
| WSP-1.2 | Module size | 41,479 | 68.4 |
| WSP-1.3 | Maximum distance wolves and sheep can move per timestep | 2821 | 28.1 |
| GoL-2.1 | Model total area | 13,765 | 5.3 |
| GoL-2.2 | Module size | 0† | 0 |
| GoL-2.3 | Number of neighbouring cells any one cell can interact with | 96 | 48.0 |
| LC-3.1 | Number of network nodes | 0 | 0 |
| LC-3.2 | Number of inter-nodal connections | 0 | 0 |
| LC-3.3 | Standard deviation of connections measured from experiment 3.2. | 0 | 0 |
| LCH-4 | Model total area | 2249 | 45.0 |
| SH-5.1 | Carrying capacity for phytoplankton (i.e. model size) | 7 | 6.9 |
| SH-5.2 | Fraction of volume exchanged between model parts (i.e. diffusion of stress) | 15 | 14.9 |

# Supplementary Figures

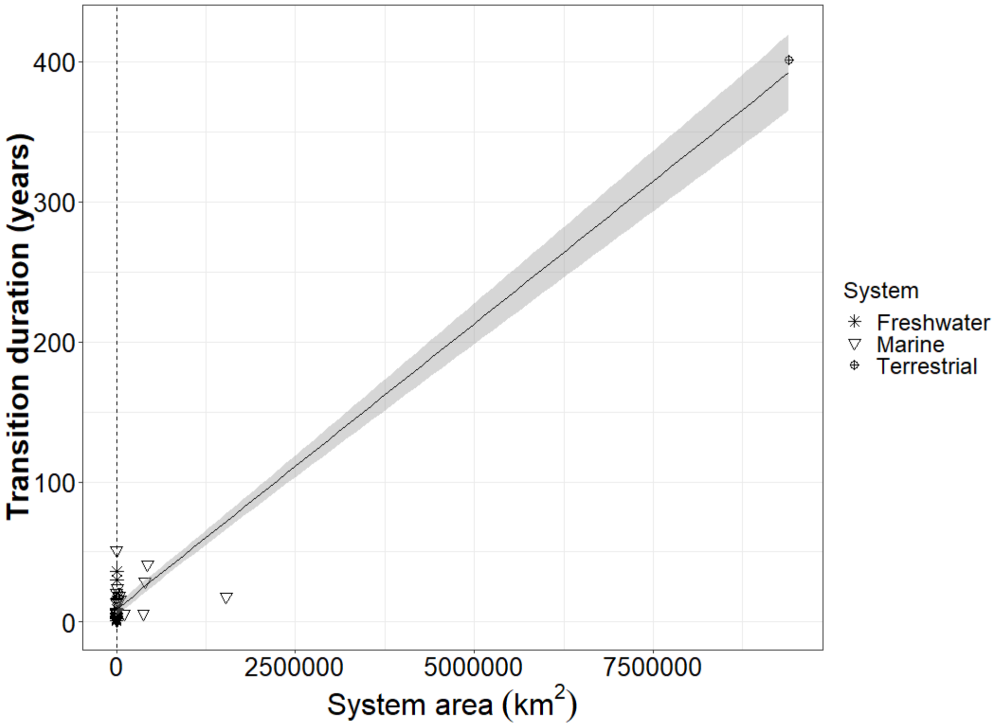


**Supplementary Figure 1|** The unlogged, linear relationship between system area and transition duration**.** The shaded region equals the standard error around the regression line for the 42 empirical data points ($R^2 = 0.95$, $p < 0.001$, df = 40).

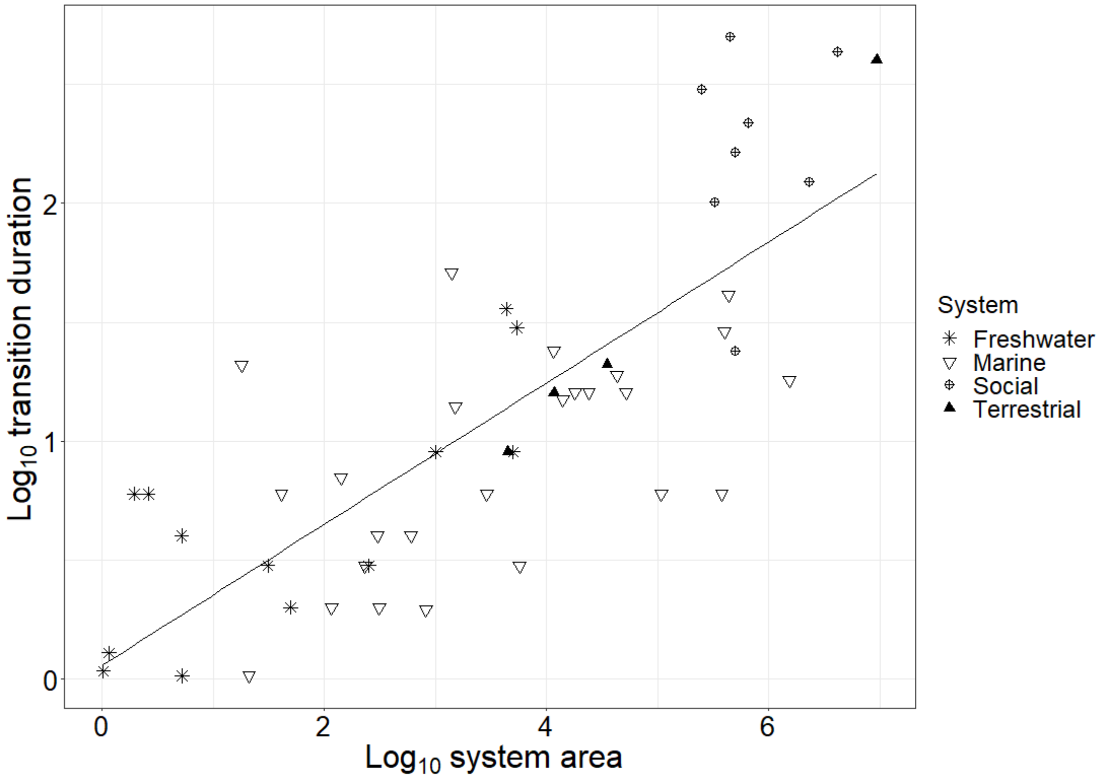


**Supplementary Figure 2|** The log-log linear relationship between the spatial extent and temporal duration of regime shifts observed in nature and 8 historical societal collapses. The relationship is significantly positive ($R^2$ = 0.607, $p < 0.05$, df = 48) and sub-linear (slope = 0.297), providing tentative evidence that relative to shifts in nature, the collapse of social systems become disproportionately slower with increasing system area. The untransformed unit of the x-axis is *kilometres-squared*, whilst the y-axis is *years.*

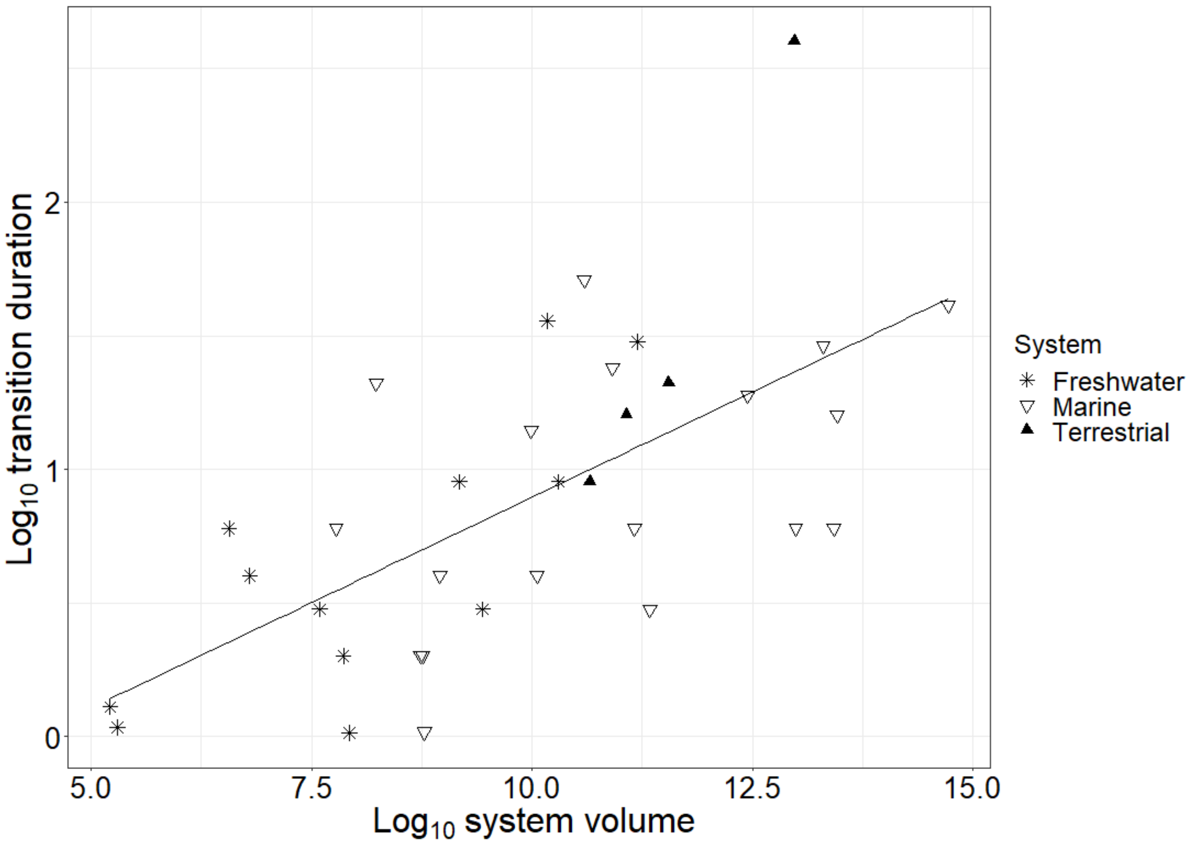


**Supplementary Figure 3|** The log-log linear relationship between the system volume and temporal duration of regime shifts observed in nature. System volume exhibits a significant positive ($R^2$ = 0.404, $p < 0.05$, df = 32) and sub-linear power law relationships (slope = 0.158) with shift duration. Only the ecosystems with volumes that could be reliably estimated are shown. The original x-axis unit was *million cubic-metres*, whilst the y-axis unit was *years.*

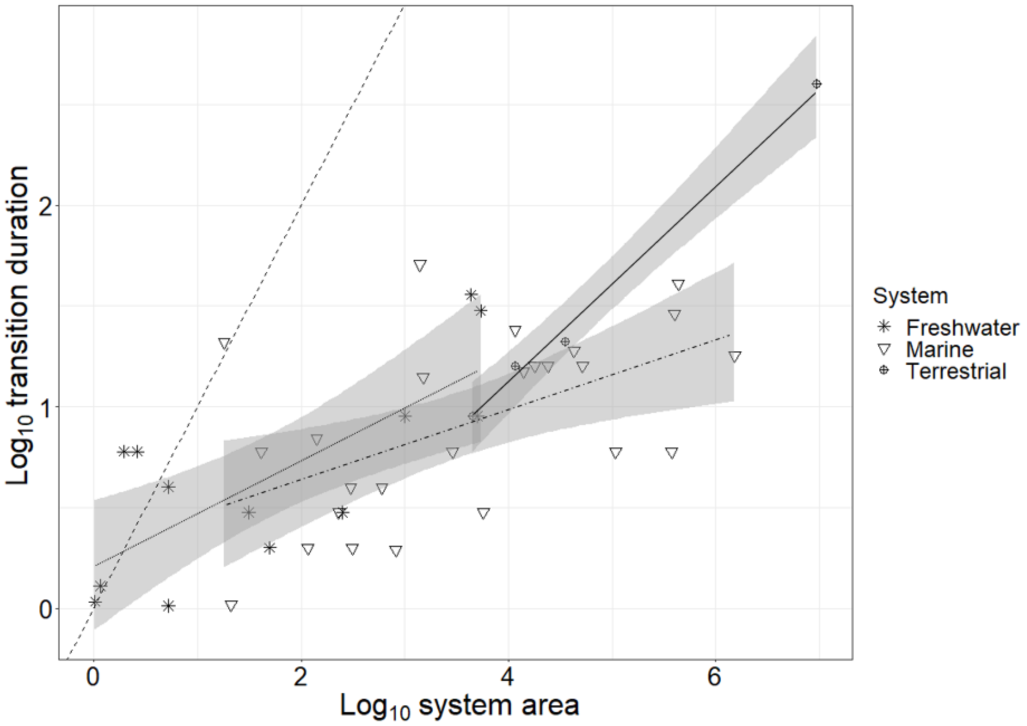


**Supplementary Figure 4|** The log-log linear relationship between the spatial extent and temporal duration of regime shifts observed in nature by system type. The empirical dataset presented in Fig. 1 with the individual regression models grouped by system type. Consistent with the overall trend presented in Fig. 1, all three of the regression models exhibit significant positive sublinear trends ($p < 0.05$). Freshwater (dotted line) – $R^2 = 0.543$, slope = 0.262, $p < 0.05$, df = 11); Marine (dot dash line) – $R^2 = 0.267$, slope = 0.174, $p < 0.05$, df = 23; Terrestrial (solid line) – $R^2 = 0.996$, slope = 0.495, $p < 0.05$, df = 2. The dashed line represents the 1:1 reference line.

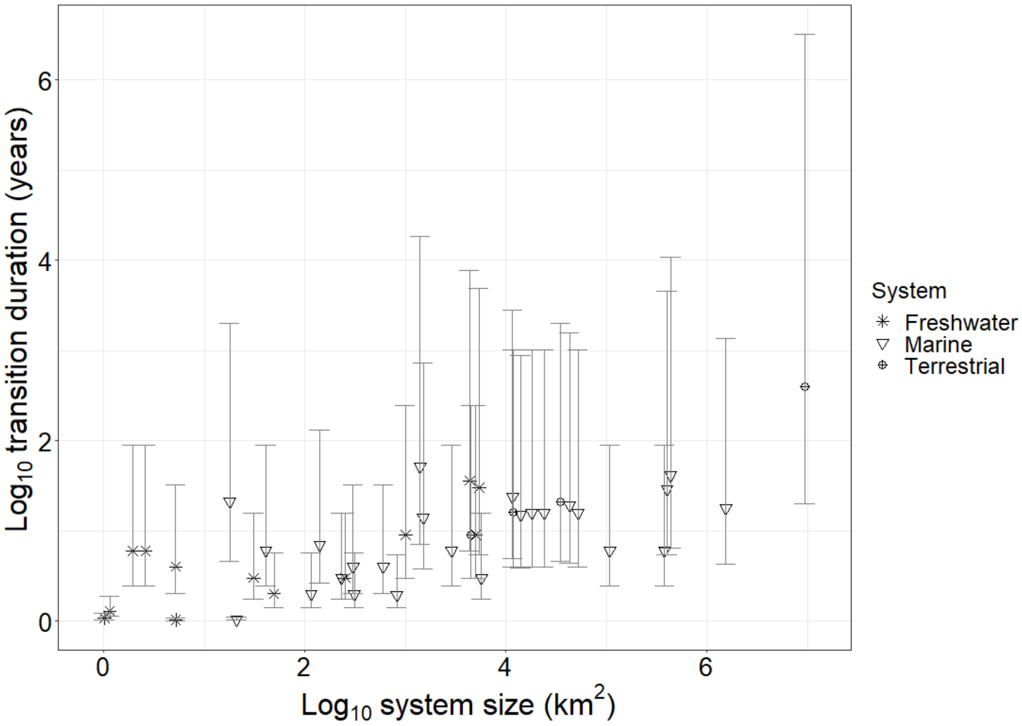


**Supplementary Figure 5|** The empirical dataset depicted with the 50-150% ranges resulting from the Monte Carlo sensitivity analysis described in the Methods section (n = 210,000; equal to 42 empirical records multiplied by 5000 error ranges). The lower ranges of the error bars were created by multiplying the empirical values by 0.5, whilst the upper ranges were created by multiplying the empirical ranges by 1.5 (see Methods for more details).

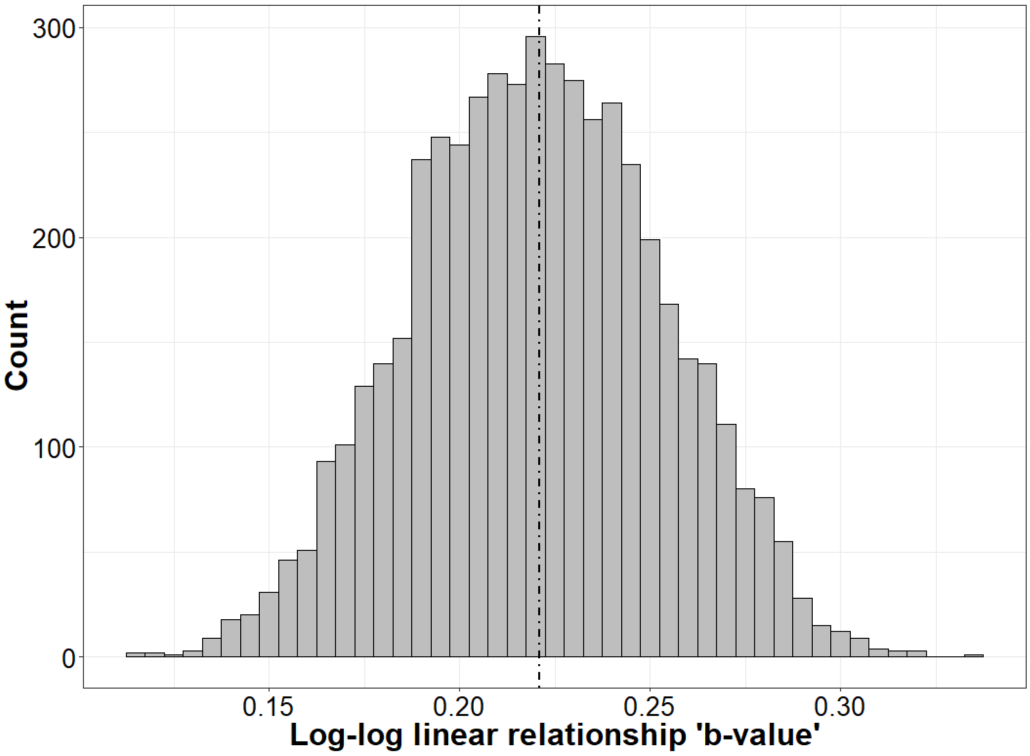


**Supplementary Figure 6|** Histogram of the b-terms (regression slope values) resulting from the production of 5,000 Monte Carlo simulations where the shift durations depicted in the original empirical model were multiplied by random error ranges between 50-150% (see Supplementary Fig. 4). The dashed line represents the median b-value of the 5,000 simulations.

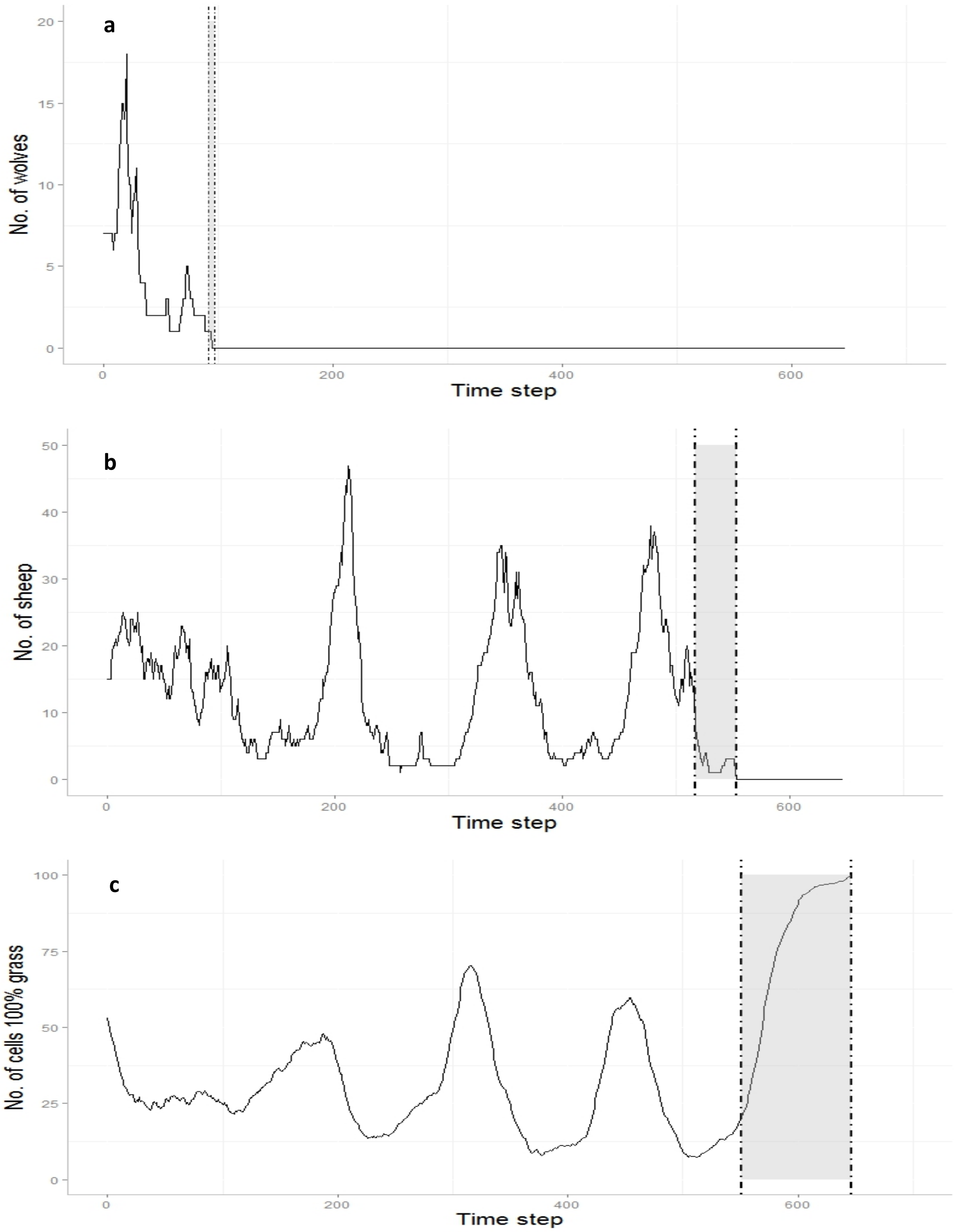


**Supplementary Figure 7|** Visualisation of the trends and regime shifts (shaded regions) of (a) wolves, (b) sheep and (c) grass, for a single run of the WSP model within the world size experiments. The dashed lines represent the starts and finishes of the respective shifts. The starts of all shifts are identified by the breakpoint function described in Methods. The end of the wolves and sheep shifts are when their respective abundances equal 0; the end of the grass shift is once the entire world area is covered by grass. Here, the corresponding shift lengths are: 3, 30, 57 (measured in time-steps).

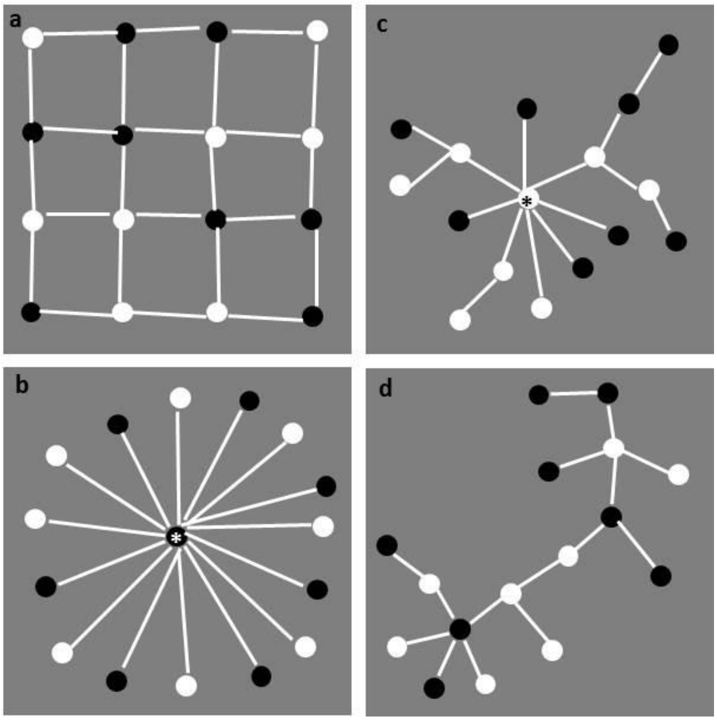


**Supplementary Figure 8|** Four LC model structures comprised of 16 nodes but with different connection distributions. The most heterogeneously wired system (*b*) has the greatest variation in the number of connections between nodes (standard deviation = 3.389, as opposed to 0.707, 1.759 and 1.183 for networks *a, c* and *d,* respectively). The asterisks mark the keystone nodes of the two most heterogeneously wired networks.

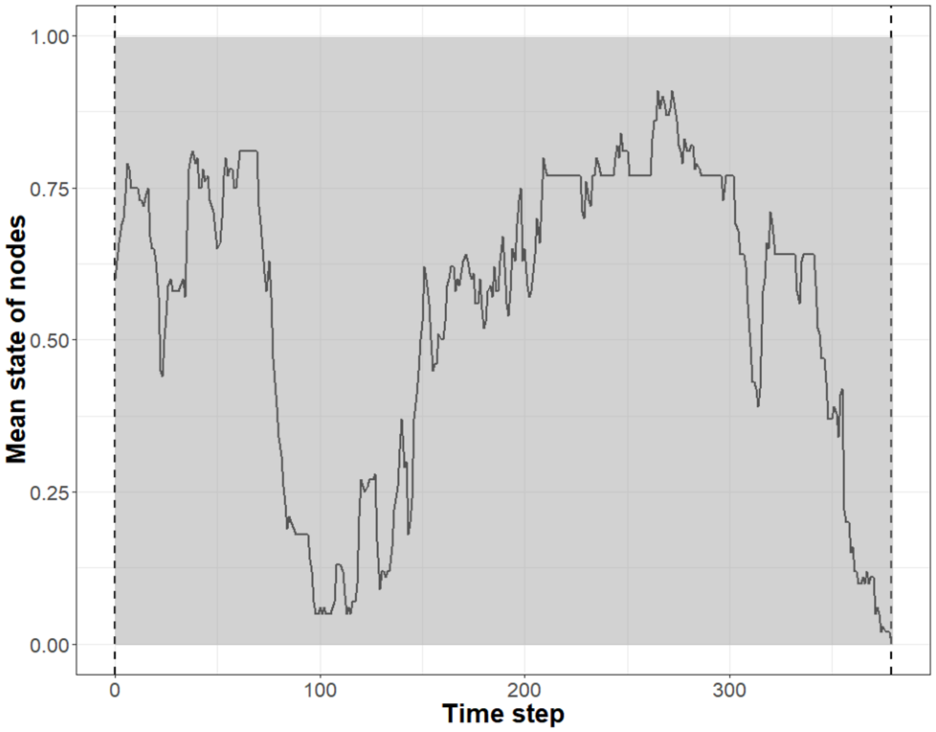


**Supplementary Figure 9|** Visualisation of the timeseries trend and shift (grey shaded) from a representative run of the LC model (Methods). As the model starts unstable, the shift is measured from the first timestep until the timestep when all nodes have the same language. The same strategy to measure the duration of shift is used for the GoL model (Methods).

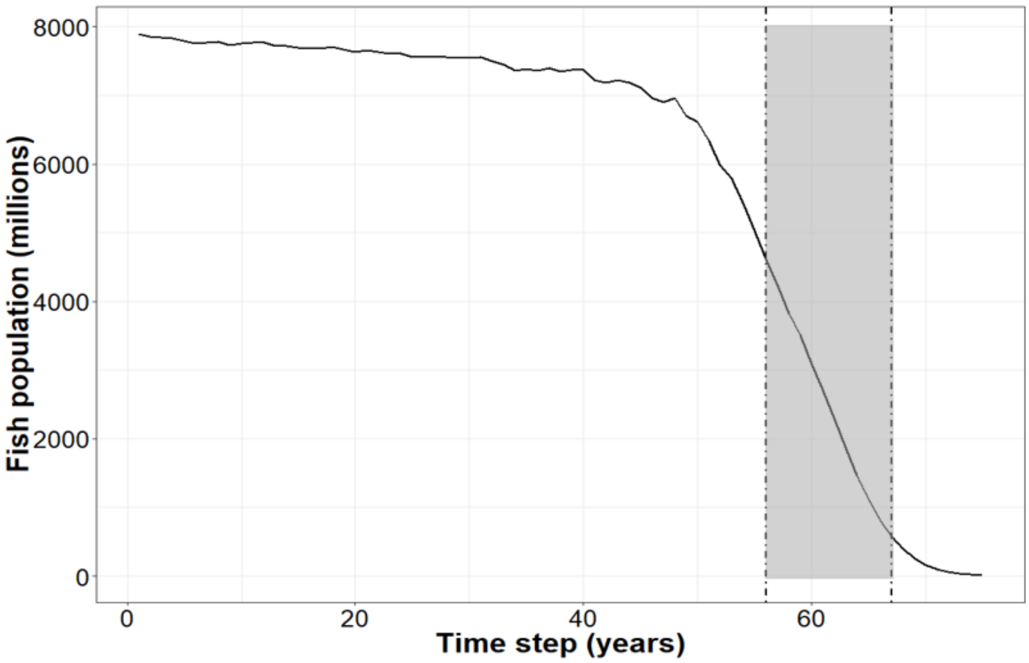


**Supplementary Figure 10|** Visualisation of the fish population trend and shift (grey shaded) from a representative run of the LCH model (Methods). The first dashed line represents the onset of the shift, as detected by the breakpoint function (Methods), and the second dashed line represents the end of the shift (i.e. fish population is less than 1% of the original fish population).

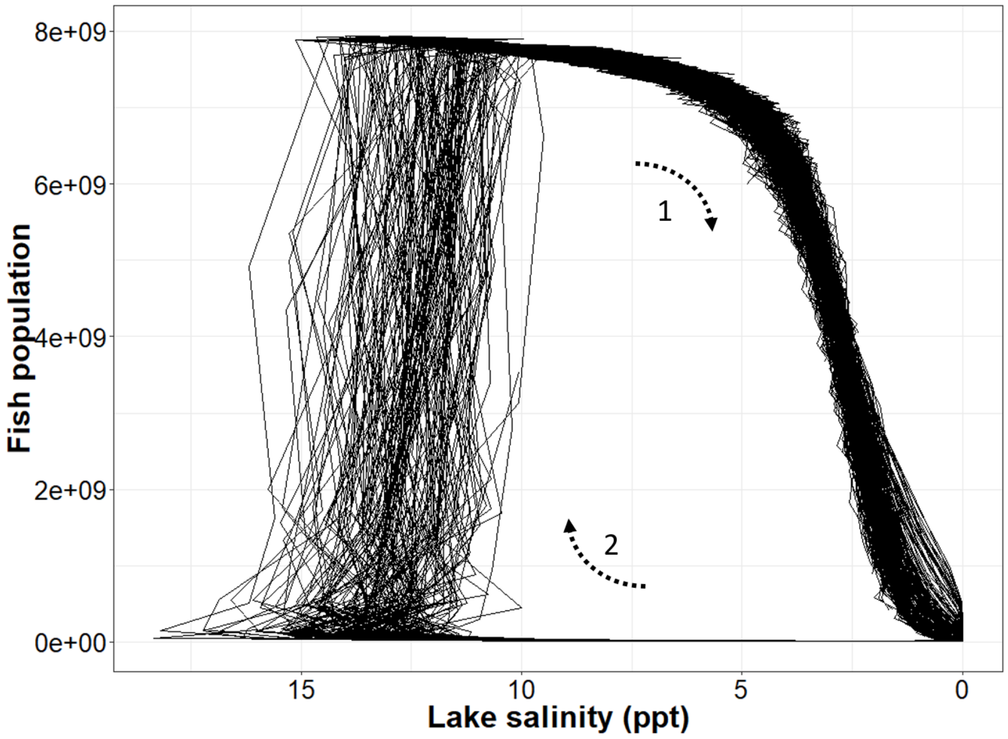


**Supplementary Figure 11|** Visualisation of the hysteresis displayed by the LCH model (note the x-axis has been reversed). The fish population collapses from left to right (dashed arrow number 1) as lake water salinity declines below ~5 parts per trillion (ppt). The recovery is stimulated by opening the tidal outlet (dashed arrow number 2); however, the recovery does not occur until lake salinity has been increased to >5 ppt past the point associated with the initial collapse. The figure shows 100 random model runs that displayed hysteresis.

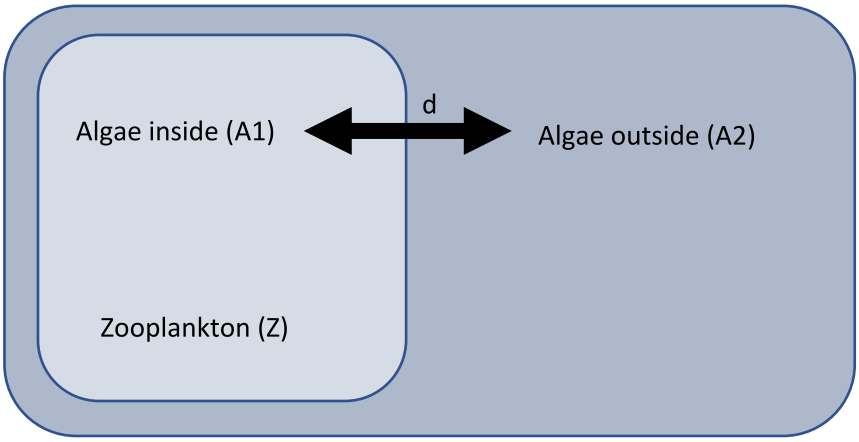


**Supplementary Figure 12|** Simplification of spatial complexity in the SH model (see Methods)

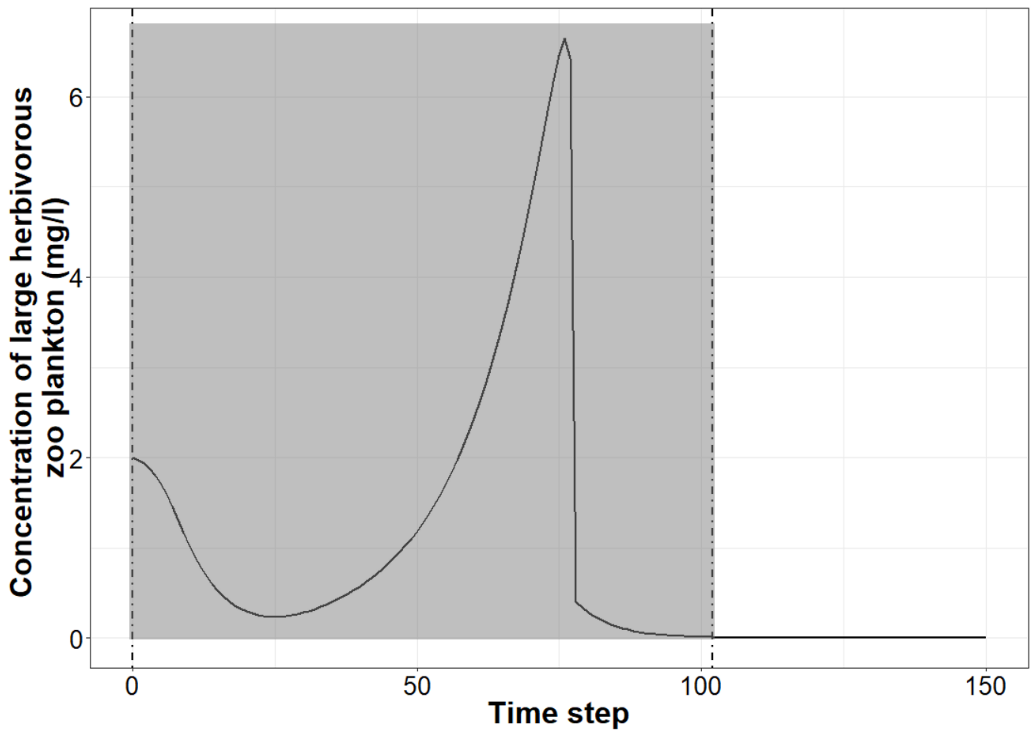


**Supplementary Figure 13|** Visualisation of the timeseries trend and shift (grey shaded) from a representative run of the SH model (Methods). As the model starts unstable, the shift is measured from the first timestep until the timestep when the concentration of zooplankton equals zero.

## Supplementary References


1. R. *R: A language and environment for statistical computing. R Foundation for Statistical Computing*. (2008).
2. Kabat, P. *et al. Vegetation, Water, Humans and the Climate*. (Springer, 2004).
3. Wang, R. *et al.* Flickering gives early warning signals of a critical transition to a eutrophic lake state. *Nature* **492**, 419–422 (2012).
4. Elser, J. J., Elser, M. M., MacKay, N. A. & Carpenter, S. R. Zooplankton-mediated transitions between N- and P-limited algal growth1. *Limnol. Oceanogr.* **33**, 1–14 (2018).
5. Carpenter, S. R. *et al.* Early Warnings of Regime Shifts: A Whole-Ecosystem Experiment. *Science (80-. ).* **332**, 1079–1082 (2011).
6. Mwita, C. & Nkwengulila, G. *Parasites of Clarias gariepinus (Burchell, 1822) (Pisces: Clariidae) from the Mwanza Gulf, Lake Victoria*. *Tanzania Journal of Science* **30**, (2005).
7. Wanink, J. H., Katunzi, E. F. B., Goudswaard, K. P. C., Witte, F. & van Densen, W. L. T. The shift to smaller zooplankton in Lake Victoria cannot be attributed to the 'sardine' Rastrineobola argentea (Cyprinidae). *Aquat. Living Resour.* **15**, 37–43 (2002).
8. Hargeby, A., Andersson, G., Blindow, I. & Johansson, S. Trophic web structure in a shallow eutrophic lake during a dominance shift from phytoplankton to submerged macrophytes. *Hydrobiologia* **279**, 83–90 (1994).
9. Bižić-Ionescu, M., Amann, R. & Grossart, H.-P. Massive Regime Shifts and High Activity of Heterotrophic Bacteria in an Ice-Covered Lake. *PLoS One* **9**, e113611 (2014).
10. Dokulil, M. Photoautotrophic productivity in eutrophic ecosystems. in *Eutrophication: Causes, Consequences and Control* (eds. Ansari, A. . & Gill, S. .) 99–110 (Springer, 2014).
11. Ndebele-Murisa, M. R., Mashonjowa, E. & Hill, T. The implications of a changing climate on the Kapenta fish stocks of Lake Kariba, Zimbabwe. *Trans. R. Soc. South Africa* **66**, 105–119 (2011).
12. Rühland, K., Paterson, A. M. & Smol, J. P. Hemispheric-scale patterns of climate-related shifts in planktonic diatoms from North American and European lakes. *Glob. Chang. Biol.* **14**, 2740–2754 (2008).
13. Robert Stone, J. & Fritz, S. C. Three-dimensional modeling of lacustrine diatom habitat areas: Improving paleolimnological interpretation of planktic : benthic ratios. *Limnol. Oceanogr.* **49**, 1540–1548 (2004).
14. Cooper, G. S. & Dearing, J. A. Modelling future safe and just operating spaces in regional social-ecological systems. *Sci. Total Environ.* **651**, 2105–2117 (2019).
15. Diaz, R. J. & Rosenberg, R. Spreading Dead Zones and Consequences for Marine Ecosystems. *Science (80-. ).* **321**, 926–929 (2008).
16. Morris, K., C. Bailey, P., I. Boon, P. & Hughes, L. *Alternative stable states in the aquatic vegetation of shallow urban lakes. II. Catastrophic loss of aquatic plants consequent to nutrient enrichment*. *Marine and Freshwater Research* **54**, (2003).

17. Hughes, T. P. Catastrophes, Phase Shifts, and Large-Scale Degradation of a Caribbean Coral Reef. *Science (80-. ).* **265**, 1547 LP – 1551 (1994).

18. Spalding, M. ., Ravilious, C. & Green, E. . *World Atlas of Coral Reefs*. (UNEP-WCMC by the University of California Press, 2001).

19. Oguz, T. & Gilbert, D. Abrupt transitions of the top-down controlled Black Sea pelagic ecosystem during 1960–2000: Evidence for regime-shifts under strong fishery exploitation and nutrient enrichment modulated by climate-induced variations. *Deep Sea Res. Part I Oceanogr. Res. Pap.* **54**, 220–242 (2007).

20. J Anderson, P. Pandalid Shrimp as Indicators of Ecosystem Regime Shift. *J. Northwest Atl. Fish. Sci.* **27**, 1–18 (2000).

21. Anderson, P. . & Piatt, J. . Community reorganization in the Gulf of Alaska following ocean climate regime shift. *Mar. Ecol. Prog. Ser.* **189**, 117–123 (1999).

22. Turner, R. E., Rabalais, N. N., Swenson, E. M., Kasprzak, M. & Romaire, T. Summer hypoxia in the northern Gulf of Mexico and its prediction from 1978 to 1995. *Mar. Environ. Res.* **59**, 65–77 (2005).

23. Yletyinen, J. Northern Gulf of Mexico. *Regime Shift DataBase* (2011). Available at: http://www.regimeshifts.org/what-is-a-regime-shift/item/133-northern-gulf-of-mexico. (Accessed: 27th September 2016)

24. Gunderson, L. H. South Florida: The reality of change and prospects for sustainability: Managing surprising ecosystems in southern Florida. *Ecol. Econ.* **37**, 371–378 (2001).

25. Yletyinen, J. Florida Bay, USA. *Regime Shift DataBase* (2012). Available at: http://www.regimeshifts.org/what-is-a-regime-shift/item/157-florida-bay-usa. (Accessed: 15th September 2016)

26. Petersen, J. K. *et al.* Regime shift in a coastal marine ecosystem. *Ecol. Appl.* **18**, 497–510 (2008).

27. van Nes, E. H., Amaro, T., Scheffer, M. & Duineveld, G. Possible mechanisms for a marine benthic regime shift in the North Sea. *Mar. Ecol. Prog. Ser. 330* **330**, 39–47 (2007).

28. Möllmann, C. & Diekmann, R. Chapter 4 - Marine Ecosystem Regime Shifts Induced by Climate and Overfishing: A Review for the Northern Hemisphere. in *Advances in Ecological Research* (eds. Guy Woodward, U. J. & Eoin, J. O.) **Volume 47**, 303–347 (Academic Press, 2012).

29. Conversi, A. *et al.* The Mediterranean Sea Regime Shift at the End of the 1980s, and Intriguing Parallelisms with Other European Basins. *PLoS One* **5**, e10633 (2010).

30. Mozetič, P. METABASE Explorer: The Marine Ecological Time-series Database. (2015). Available at: http://www.st.nmfs.noaa.gov/copepod/time-series/si-10101/. (Accessed: 26th June 2016)

31. Möllmann, C. *et al.* Reorganization of a large marine ecosystem due to atmospheric and anthropogenic pressure: a discontinuous regime shift in the Central Baltic Sea. *Glob. Chang. Biol.* **15**, 1377–1393 (2009).

32. Bonsdorf, E., Rönnberg, C. & Aarnio, K. Some ecological properties in relation to

eutrophication in the Baltic Sea. *Hydrobiologia* **475476**, 371–377 (2002).

33. Giri, C. P. & Long, J. Mangrove reemergence in the northernmost range limit of eastern Florida. *Proc. Natl. Acad. Sci.* **111**, E1447 LP-E1448 (2014).

34. Hamilton, L. C. & Butler, M. J. Outport Adaptations: Social Indicators through Newfoundland's Cod Crisis. *Res. Hum. Ecol.* **8**, 11 (2001).

35. Boyer, D. C. & Hampton, I. An overview of the living marine resources of Namibia. *South African J. Mar. Sci.* **23**, 5–35 (2001).

36. Glaspie, C. N., Seitz, R. D., Ogburn, M. B., Dungan, C. F. & Hines, A. H. Impacts of predators, habitat, recruitment, and disease on soft-shell clams Mya arenaria and stout razor clams Tagelus plebeius in Chesapeake Bay. *Mar. Ecol. Prog. Ser.* **603**, 55 (2018).

37. Biggs, R. O. Chesapeake Bay. *Regime Shift DataBase* (2012). Available at: https://regimeshifts.org/component/k2/item/461-chesapeake-bay. (Accessed: 20th September 2016)

38. SIF. *Aldabra atoll, Seychelles*. (2002).

39. Stobart, B., Teleki, K., Buckley, R., Downing, N. & Callow, M. Coral recovery at Aldabra Atoll, Seychelles: five years after the 1998 bleaching event. *Philos. Trans. R. Soc. London A Math. Phys. Eng. Sci.* **363**, 251–255 (2005).

40. Grantham, B. A. *et al.* Upwelling-driven nearshore hypoxia signals ecosystem and oceanographic changes in the northeast Pacific. *Nature* **429**, 749 (2004).

41. Beuchel, F., Gulliksen, B. & Carroll, M. . Long-term patterns of rocky bottom macrobenthic community structure in an Arctic fjord (Kongsfjorden, Svalbard) in relation to climate variability (1980–2003). *J. Mar. Syst.* **63**, 35–48 (2006).

42. Walkusz, W. *et al.* Characteristics of the Arctic and Antarctic mesozooplankton in the neritic zone during summer. *Polish Polar Res.* **25**, 275–291 (2004).

43. Balkis, N. *The Effect of Marmara (Izmit) Earthquake on the Chemical Oceanography and Mangan Enrichment in the Lower Layer Water of Izmit Bay, Turkey*. (2012). doi:10.5772/27939

44. Winding Hansen, B., Stenalt, E., Petersen, J. & Ellegaard, C. *Invertebrate re-colonisation in Mariager Fjord (Denmark) after severe hypoxia. I. Zooplankton and settlement*. *Ophelia* **56**, (2002).

45. R N Gibson *et al. Temporal and spatial large-scale effects of eutrophication and oxygen deficiency on benthic fauna in Scandinavian and Baltic waters-a review*. *Annual Review* **40**, (2002).

46. Brodziak, J., Traver, M. & Smith, L. *The nascent recovery of the Georges Bank haddock stock*. *Fisheries Research* **94**, (2008).

47. Mann, K. . Destruction of kelp-beds by sea-urchins: A cyclical phenomenon or irreversible degradation? *HelgolS.nder wiss. Meeresunters* **30**, 455–467 (1977).

48. Arias Schreiber, M. & Halliday, A. Uncommon among the Commons? Disentangling the Sustainability of the Peruvian Anchovy Fishery. *Ecol. Soc.* **18**,

49. Hylleber, J. *Extinction and immigration of benthic fauna: the value of historical data from Limfjorden, Denmark*. (1993).

50. Yasuhara, M. & Yamazaki, H. The impact of 150 years of anthropogenic pollution on the shallow marine ostracode fauna, Osaka Bay, Japan. *Mar. Micropaleontol.* **55**, 63–74 (2005).

51. deMenocal, P. *et al.* Abrupt onset and termination of the African Humid Period:: rapid climate responses to gradual insolation forcing. *Quat. Sci. Rev.* **19**, 347–361 (2000).

52. Tshimpanga, J.M Peterson, G., Biggs, R. & Enfors, E. Maradi Agro-ecosystem. *Regime Shift DataBase* (2011). Available at: http://www.regimeshifts.org/about/item/57-maradi-agro-ecosystem. (Accessed: 11th June 2016)

53. Ripple, W. J. & Beschta, R. L. Linking a cougar decline, trophic cascade, and catastrophic regime shift in Zion National Park. *Biol. Conserv.* **133**, 397–408 (2006).

54. Meyers, J. Grassland to woodland, Chobe National Park, Botswana. *Regime Shift DataBase* Available at: http://www.resalliance.org/tdb-database/12. (Accessed: 10th June 2016)

55. Taagepera, R. Size and Duration of Empires: Growth-Decline Curves, 600 B.C. to 600 A.D. *Soc. Sci. Hist.* **3**, 115–138 (1979).

56. Taagepera, R. Growth curves of empires. *Int. J. Gen. Syst.* **13**, 171–176 (1968).

57. Reyes-Foster, B. Maya Geography and Culture: Ancient and Contemporary. in *Encyclopedia of Global Archaeology* (ed. Smith, C.) 4710–4715 (Springer New York, 2014). doi:10.1007/978-1-4419-0465-2_2041

58. Taagepera, R. Expansion and Contraction Patterns of Large Polities: Context for Russia. *Int. Stud. Q.* **41**, 475–504 (1997).
