# Supplementary material for: Regime shifts occur disproportionately faster in larger ecosystems
Source: Nat Commun. 2020 Mar 10;11:1175. doi: 10.1038/s41467-020-15029-x (PMC7064493; doi:10.1038/s41467-020-15029-x)
Supplement: Supplementary file 3 — Reporting Summary [file 41467_2020_15029_MOESM3_ESM.pdf]

nature research

Corresponding author(s): John Dearing

Last updated by author(s): Feb 13, 2020

Reporting Summary

Nature Research wishes to improve the reproducibility of the work that we publish. This form provides structure for consistency and transparency in reporting. For further information on Nature Research policies, see Authors & Referees and the Editorial Policy Checklist .

Statistics

For all statistical analyses, confirm that the following items are present in the figure legend, table legend, main text, or Methods section.

n/a	Confirmed	
☐	☒	The exact sample size (*n*) for each experimental group/condition, given as a discrete number and unit of measurement
☐	☒	A statement on whether measurements were taken from distinct samples or whether the same sample was measured repeatedly
☐	☒	The statistical test(s) used AND whether they are one- or two-sided<br>*Only common tests should be described solely by name; describe more complex techniques in the Methods section.*
☐	☒	A description of all covariates tested
☐	☒	A description of any assumptions or corrections, such as tests of normality and adjustment for multiple comparisons
☐	☒	A full description of the statistical parameters including central tendency (e.g. means) or other basic estimates (e.g. regression coefficient) AND variation (e.g. standard deviation) or associated estimates of uncertainty (e.g. confidence intervals)
☐	☒	For null hypothesis testing, the test statistic (e.g. *F*, *t*, *r*) with confidence intervals, effect sizes, degrees of freedom and *P* value noted<br>*Give P values as exact values whenever suitable.*
☒	☐	For Bayesian analysis, information on the choice of priors and Markov chain Monte Carlo settings
☒	☐	For hierarchical and complex designs, identification of the appropriate level for tests and full reporting of outcomes
☒	☐	Estimates of effect sizes (e.g. Cohen's *d*, Pearson's *r*), indicating how they were calculated

Our web collection on statistics for biologists contains articles on many of the points above.

Software and code

Policy information about availability of computer code

Data collection	NetLogo v5.1<br>Stella (ISEE Systems) v10.1
Data analysis	R version 3.4.4 (2018-03-15) -- "Someone to Lean On"

For manuscripts utilizing custom algorithms or software that are central to the research but not yet described in published literature, software must be made available to editors/reviewers. We strongly encourage code deposition in a community repository (e.g. GitHub). See the Nature Research guidelines for submitting code & software for further information.

Data

Policy information about availability of data

All manuscripts must include a data availability statement. This statement should provide the following information, where applicable:

- Accession codes, unique identifiers, or web links for publicly available datasets
- A list of figures that have associated raw data
- A description of any restrictions on data availability

All data generated or analysed during this study are included in this published article (and its supplementary information files).

Field-specific reporting

Please select the one below that is the best fit for your research. If you are not sure, read the appropriate sections before making your selection.

☐ Life sciences ☐ Behavioural & social sciences ☒ Ecological, evolutionary & environmental sciences

Ecological, evolutionary & environmental sciences study design

All studies must disclose on these points even when the disclosure is negative.

Study description	Desktop study of the relationship between ecosystem size and duration of regime shift. Statistical analysis of regime shift duration in published observed data for ecosystems/social systems. Simulation of regime shift durations in five open access/available models to study the relationship with system area.
Research sample	The ~50 empirical data points were collected from published sources, as per the protocol detailed in Methods. The protocol essentially established a standard to reliably measure the spatial and temporal scales of regime shifts, whilst filtering out any seasonal events (e.g. annual red tide events). The models were also selected via a second protocol outlined in the Methods. Essentially the models had to simulate state changes driven by some measurable driver (as opposed to some randomness). As a result, the model results were generated from five freely available models, written in NetLogo v5.1 and Stella system dynamics v10.1.
Sampling strategy	Used all published data available from literature search following quality control as described in Methods.
Data collection	All data collected from publications and model outputs as described in Methods
Timing and spatial scale	Timing and spatial scale set only for model runs as described in Methods
Data exclusions	The exclusion criteria were pre-established for all model runs across the five modelling experiments. Any model run that did not meet the criteria for inclusion described in the Methods (i.e. a statistically significant breakpoint or the settling within an alternative stable state) was excluded from analysis. Please see Supplementary Note 2 and Supplementary Table 9 for further details.
Reproducibility	Reproducibility exists in terms of analyses of multiple runs of each simulation model using sensitivity tests (e.g Monte Carlo). Spread of values for each model experiment shown in figures. All data and model code are shown in Supplementary Information or easily accessible.
Randomization	No randomization required
Blinding	No blinding required

Did the study involve field work? ☐ Yes ☒ No

Reporting for specific materials, systems and methods

We require information from authors about some types of materials, experimental systems and methods used in many studies. Here, indicate whether each material, system or method listed is relevant to your study. If you are not sure if a list item applies to your research, read the appropriate section before selecting a response.

Materials & experimental systems

n/a	Involved in the study
☒	☐ Antibodies
☒	☐ Eukaryotic cell lines
☒	☐ Palaeontology
☒	☐ Animals and other organisms
☒	☐ Human research participants
☒	☐ Clinical data

Methods

n/a	Involved in the study
☒	☐ ChIP-seq
☒	☐ Flow cytometry
☒	☐ MRI-based neuroimaging
